# Health Tract, No. 109

**Published:** 1865

**Authors:** 


### HEALTH TRACT, No. 109.

### WHITLOW.

It is sufficiently near the truth for general practical purposes to say, that a real genuine "whitlow" is a "boil," low down, next the bone, under the "whit-leather' —shall we say a boil under the white-leather, as the origin of the name? This ailment is generally at the ends of the fingers, inside, and is usually caused by pricks, bruises, and burns, but not always; for it has sometimes gone through whole neighborhoods, like measles, mumps, or cholera, and prevails more in winter and cold latitudes. If it is above the whit-leather or *fascia*, a whitlow causes comparatively little suffering; but most to those who, by hard work, keep the skin of the palm and fingers hard, thus making it more difficult for the "boil" to "break," that is, more difficult for the matter to make an opening and escape from the system. These get well of themselves, without leaving any permanently ill effects, if the system is kept free, if the part is kept moist and warm, and nothing is eaten for a few days but bread and water, fruits, and gruels or soups. But real whitlows, namely, where the boil is *below* the white or *whit*-leather, (*fascia*,) become a perfect and unendurable torture, and often cause the decay of the bone or the permanent loss of the use of the finger. To prevent this, and to give instantaneous, permanent, and safe relief, there is only one method which never fails. Get a physician to cut down to the bone, first in one direction and then another, making a cross, the object being to let out the pent-up matter, just as a common boil ceases to pain as soon as the skin is broken and the matter is let out. The matter of whitlow is more perfectly emptied out if, after this "crucial incision," the part is held in warm water for half an hour or more, and is then kept moist and warm by any sort of poultice; and that material is best which keeps moist the longest. There are multitudes of "remedies" for whitlow in the newspapers, every one of which, for real whitlow, is fallacious or impossible; that, for example, of tying a cord around the finger, to "starve it to death," by cutting off the supply of blood, just about equal to a tooth-drawing operation protracted during twenty-four hours! There is nearly always constipation, and the greater the constipation the greater the agony of a real whitlow; hence this should always be removed by injections, or better still, by the free use of coarse breads and fruits and berries, in any and every shape or form. The spot of a superficial whitlow or boil soon begins to turn yellow, but in the deep-seated or only real whitlow, after days and nights of intense pain and violent throbbing of the part, there is no yellowness, the skin is merely swollen or red; besides, the pain of a real whitlow seems to be down to the bone itself, deep-seated, and not near the surface.

